# The Public Analyst and the New Food and Drugs Act (1955)

**Published:** 1957-01

**Authors:** E. G. Whittle

**Affiliations:** Public Analyst and Official Agricultural Analyst for the City and County of Bristol and the County of Gloucester


### HE PUBLIC ANALYST AND THE NEW FOOD AND DRUGS ACT (1955)

BY

E. G. WHITTLE, B.SC., F.R.I.C.

*blic Analyst and Official Agricultural Analyst for the City and County of Bristol and the County of Gloucester*

### THE WORK OF A PUBLIC ANALYST

In England there are about twenty full-time officers of local authorities, and forty nsultants and part-time Analysts who deal with Food and Drug Analyses, and minations of Fertilizers and Feeding Stuffs.

In the Bristol laboratory the Analyst provides this service for Bristol and for oucestershire. The Analyst has many other duties such as the examination of waters, vage and sewage effluents, trade effluent, swimming bath samples, analyses under Rag Flock Act and the Poisons and Pharmacy Act. We are also very interested in nospheric pollution, and a tenth of our work includes rainfall readings and analyses sulphur pollution. The results are sent to the Department of Scientific and Industrial search for correlation with figures from other parts of the country. We deal h other pollution problems for the Inspectors of the Chief Sanitary Inspector's partment of the City. Problems about petrol and diesel exhaust fumes are beyond our pe. Other work relates to the control of water supplies to hospitals, the chlorination sewage and related matters, and more recently we have made spectroscopic analyses vitamins and trace metals.

We also undertake over one thousand analyses a year for a wide variety of problems luding infestation and foreign bodies in foods, biochemical and toxicological lyses primarily for hospitals, samples submitted by District Inspectors of the ief Sanitary Inspector's Department, occasional work in the past for ministries, l work for University and Corporation departments. Most of the Corporation rk is from the Port Health Office, the City Engineer, and the Central Purchasing partment, but all Corporation departments use the laboratory. The City Police and e Services use the department occasionally, but with the establishment of the South stern Forensic Science Laboratory much of the police work which was carried out the Public Analyst a few years ago is now carried out there. We also give advice and ormation to other Corporation departments, and deliver lectures and talks on il Defence and the work of the Department.

### THE FOOD AND DRUGS ACT (1955)

t is obviously dangerous for food to contain chemicals whose fate in the body is not wn. Such chemicals may be introduced accidentally, or deliberately, by manufacers, either to preserve the food or to satisfy the public's taste. Use of substances the antioxidants and antimould agents are potentially dangerous; some colouring ters used in foods are believed to be toxic and the Food Standards Committee the Ministry think the indiscriminate use of some of these should be stopped. ier matters that concern Analysts are the widespread and increasing use of toxic stances in agriculture and the use of fumigants in the storage of food, and of newer rodenticides which might find their way into foods. All these different lth hazards have made it necessary to strengthen the existing law in relation to the ition of injurious substances to food, and for devising new methods of control.

he new section 1 of the Act replaced sections 1 and 2 of the 1938 Act and now not prohibits the addition of a substance to a food, but also the use of any substance n ingredient in the preparation of food, or the subjection of food to any process or treatment which renders it injurious to health. This has removed a serious amb of the 1938 Act, inasmuch as that in deciding what is injurious to health, regard sh had to the cumulative effect of the consumption of articles of similar compo

Section 3 gives the Ministers of Food and Health wide powers to make regul to require, prohibit, or regulate the addition of substances to foods, and to regula composition of food if expedient for public health, or the protection of the publi similar powers in the control of processing and treatment of food. It is also provi this section that where a food is certified by a Public Analyst as being food to whi regulations apply, that food may be seized and condemned on a magistrate's or being unfit for human consumption.

Further, section 3 (2) states that "In exercise of their functions under this se the Ministers shall have regard to the desirability of restricting as far as practi the use of substances of no nutritional value as foods or as ingredients of fo

Section 5 creates two distinct offences regarding labelling—one for a false label second for a misleading one, and further declares that a label or advertisement wh calculated to mislead as to the nutritional or dietary value of any food, is calcula mislead as to the quality of the food.

Section 11 brings rich milks like Channel Island and South Devon within the of the Act in providing for regulations requiring a minimum of 4 per cent. of mi

Section 12 reintroduces the requirement that the word "cream" used in relat cream confectionery shall mean genuine cream and not reconstituted or imi cream. Artificial cream now has the more appropriate description of "reconst cream", whilst imitation cream is introduced and defined as that which not being or reconstituted cream, resembles cream in appearance, and is produced by emuls edible oils and fats with water, with or without substances not prohibited by regu

Under Section 13 there is to be constituted a Food Hygiene Advisory Cou consider and advise on questions of labelling, marking and advertising of questions of food hygiene and extension of registration or publication of Co Practice.

Section 20 is of great importance to Public Analysts and Sampling Officers. is a new procedure for disposal of samples taken for analysis, and it is now pres that division of the sample comes first and the information of intent is given on ha over the part of the sample. Detailed instructions are given as to who shall recei second part of the sample according to conditions.

Section 21 is likely to prove extremely useful in dealing with the sampling opened containers, for it is now permissible to divide such containers into lots if division is not reasonable or practicable. This means that, if for example, three a commodity were purchased, each unopened can could form part of the sample. I hold very strong views on the validity of this method of sampling, consider it can only apply if the cans carry the same batch number. I would like all pre-packed commodities bearing a date of manufacture. I see no practical diffi in this, and such dating would be of great help to the housewife, and indeed I fe has a right to know the age of the commodities she purchases. Such a scheme also lead to better "housekeeping" and turnover of goods in stores. The sug division of unopened cans is, however, not intended to apply to articles sold loos as meat pies where divisions into parts has proved difficult. Indeed it has been nec to take a balance to the shop in order to subdivide crust, meat and jelly into w equal parts. Nevertheless there remain a number of commodities which can divided and the section will hence be of value.

Section 21 (2) states that in regulations made to control compensation and lab methods of analysis may be laid down, and if so, evidence of analysis by the pres test shall be *preferred* to that of any other test. Some methods have alread prescribed in Food Standards Orders, for example, vitamin A in margarine. Public Analysts have the task of making methods work, it is to be hoped that the be fully consulted before such tests are incorporated in regulations.

Section 21 (3) provides a long-overdue relaxation in permitting the analysis to be made by any person acting under the direction of the Public Analyst, who nevertheless must, as always, sign the certificate.

Section 21 (4) permits the Public Analyst to pass on a sample to another Public Analyst if he himself is not in a position to do a particular type of analysis, for example, where specialized equipment is necessary.

Section 25 lays down the time limit for prosecutions. For milk samples this remains 28 days, but is increased to 2 months for food and drugs. On a magistrate's order the limit may be extended to 42 days for milk, and indefinitely for other articles. Since the labelling offences the Ministry of Agriculture, Fisheries and Food now requires 14 days' notice of intention to prosecute, the actual available analytical time is reduced to six weeks.

Section 26 increases penalties for offences under the Act to a fine of £100 or 3 months' imprisonment, or both, with a fine of £5 per day for continuing offences.

The definition of analysis now includes microbiological assay but no other form of biological assay. This leaves the full meaning of analysis still undefined.

The definition of food now includes chewing gum and other products of a like nature, but does not include live animals and birds, and articles or substances used *only* as drugs.

Finally to strengthen the Act in "unfit for human consumption" clauses, the definition of human consumption includes "the use in the preparation of food for human consumption."

It will be seen that there is nothing drastic in the new Act, but it stops a number of loopholes, and what is even more important, it gives the Minister powers to exercise much stricter control of accidental or deliberate chemical additions and of hygiene in relation to the preparation of food.

